# A mini review of genome-predicted β-lactam susceptibility in *Streptococcus pneumoniae*: from PBP profiles to potential clinical interpretation

**DOI:** 10.3389/fcimb.2026.1901030

**Published:** 2026-07-22

**Authors:** Ziyi Yan, Li Liu, Yanying Ren, Jiaji Ling, Jing Liao, Xingxin Liu, Yingying Li, Xia Wang, Linghan Kuang, Wei Zhou, Yongmei Jiang, Yali Cui

**Affiliations:** 1 Department of Laboratory Medicine, West China Second University Hospital, Sichuan University, Chengdu, China; 2 Key Laboratory of Birth Defects and Related Diseases of Women and Children of Ministry of Education, West China Second University Hospital, Sichuan University, Chengdu, China; 3 Department of Blood Transfusion, Beijing Anzhen Nanchong Hospital of Capital Medical University & Nanchong Central Hospital, Nanchong, China; 4 Department of Medical Laboratory, Dazhou Integrated Traditional Chinese Medicine & Western Medicine Hospital, Dazhou Second People’s Hospital, Dazhou, China; 5 Department of Laboratory Medicine, Tibet Autonomous Region Women’s and Children’s Hospital, Lhasa, China; 6 Department of Laboratory Medicine, Chengdu Hi-Tech Zone Hospital for Women and Children (Chengdu Hi-Tech Zone Hospital for Maternal and Child Healthcare), Chengdu, China; 7 Department of Laboratory Medicine, West China Second University Hospital (Tianfu), Sichuan University (Sichuan Provincial Children’s Hospital), Meishan, China; 8 Department of Laboratory Medicine, Meishan Women and Children’s Hospital, Alliance Hospital of West China Second University Hospital, Sichuan University, Meishan, China

**Keywords:** antimicrobial susceptibility, minimum inhibitory concentration, penicillin-binding proteins, *Streptococcus pneumoniae*, whole-genome sequencing, β-lactam susceptibility

## Abstract

β-Lactams remain central to the treatment of pneumococcal infections, and whole-genome sequencing increasingly enables pneumococcal β-lactam susceptibility to be inferred from the combined transpeptidase-domain sequences of PBP1a, PBP2b, and PBP2x. PBP-profile lookup, statistical models, and integrated genomic pipelines can predict drug-specific minimum inhibitory concentrations with high overall agreement with phenotypic antimicrobial susceptibility testing. However, technical prediction accuracy does not by itself establish direct clinical use. Novel or sparsely represented PBP profiles, interspecies recombination within the mitis-group gene pool, lineage and geographical structure, non-PBP genetic effects, and uncertainty in reference MIC measurements can limit model transportability. Furthermore, a predicted MIC cannot be converted into a clinically meaningful susceptibility interpretation without considering the antimicrobial agent, infection site, dosing or exposure context, interpretive standard, and breakpoint version. This mini review summarizes the PBP-centered genetic architecture of pneumococcal β-lactam susceptibility, evaluates current approaches for genome-based MIC prediction, and examines the factors that constrain their generalizability. We propose a three-layer reporting framework that separates genomic findings, predicted phenotypes, and potential clinical interpretation, while explicitly communicating prediction confidence and identifying circumstances requiring confirmatory phenotypic MIC testing. Genome-based PBP profiling is already well positioned to strengthen pneumococcal surveillance and may inform potential clinical interpretation, but patient-level reporting will require continuously curated phenotype-linked databases, external validation in intended-use populations, and an uncertainty-aware interpretive layer connecting genomic evidence to treatment-specific breakpoints.

## 1 Introduction

β-Lactams remain central to the treatment of pneumococcal infections, but genome-based inference of their activity against *Streptococcus pneumoniae* (*S. pneumoniae*) differs fundamentally from the detection of a single acquired resistance gene. Reduced susceptibility arises predominantly from mosaic variation in the transpeptidase domains of PBP1a, PBP2b, and PBP2x, and the combined sequence context of these proteins correlates strongly with drug-specific minimum inhibitory concentrations (MICs). This relationship established the basis for using PBP profiles to predict β-lactam susceptibility from whole-genome sequencing data (Li et al., 2016).

Over the past decade, PBP-profile lookup, statistical models, and integrated genomic platforms have progressively moved this approach from proof of concept toward scalable application. In a recent direct comparison with conventional antimicrobial susceptibility testing, Pathogenwatch and AREScloud achieved greater than 94% categorical agreement for the evaluated β-lactams (Sanchez et al., 2024). However, technical concordance alone does not establish that genome-based predictions can be used directly for patient-level clinical interpretation. Whole-genome sequencing-based susceptibility prediction requires curated phenotype-linked databases, standardized quality control, and validation within the intended application (Samuelsen et al., 2026). Moreover, a predicted MIC is not a context-independent treatment recommendation: clinical breakpoints incorporate microbiological and pharmacokinetic/pharmacodynamic evidence and are linked to specific antimicrobial agents, dosing regimens, and therapeutic settings (Mouton et al., 2012).

This mini review examines the pathway from pneumococcal PBP sequences to potential clinical interpretation of β-lactam susceptibility. We first summarize the PBP-centered genetic architecture underlying β-lactam MIC variation, then review current prediction approaches and the factors limiting their generalizability. Finally, we propose a three-layer framework that separates genomic findings, predicted phenotypes, and clinical interpretation, and identify the circumstances in which confirmatory phenotypic MIC testing remains necessary. Rather than positioning genomic prediction as an immediate replacement for phenotypic testing, this review defines the evidence and interpretive steps required before genomic prediction can be considered for clinical reporting.

## 2 The PBP-centered genetic architecture of β-lactam susceptibility

β-Lactams inhibit pneumococcal cell-wall synthesis by acylating the transpeptidase domains of penicillin-binding proteins (PBPs), thereby impairing peptidoglycan cross-linking. In *Streptococcus pneumoniae*, reduced β-lactam susceptibility is mediated predominantly by structurally altered, low-affinity PBPs rather than by β-lactamase production. PBP2x and PBP2b act as major primary determinants, while altered PBP1a commonly contributes to the development of higher-level resistance after changes in other PBPs have been acquired (Grebe and Hakenbeck, 1996; Gibson et al., 2022). Nevertheless, resistance cannot be reliably inferred from a short list of individual substitutions. Resistant *pbp* alleles are frequently mosaic and contain multiple linked amino-acid changes, whose phenotypic effects depend on the affected PBP, the β-lactam tested, and the accompanying alleles. Consistent with this combinatorial architecture, the joint transpeptidase-domain sequences of PBP1a, PBP2b, and PBP2x—represented as a PBP profile—explain substantially more variation in β-lactam MICs than isolated mutations and provide the principal genomic basis for MIC prediction (Li et al., 2016).

The emergence of these profiles is an evolutionary and path-dependent process. As a naturally competent and highly recombinogenic species, *S. pneumoniae* can acquire divergent *pbp* alleles from other pneumococci and related mitis-group streptococci, generating mosaic loci through homologous recombination. Population-genomic analyses have demonstrated that interspecies recombination has repeatedly modified resistance-associated core loci during the emergence of antibiotic-resistant pneumococcal lineages (D'Aeth et al., 2021). Experimental reconstruction of amoxicillin resistance further showed that clinically relevant resistance required the acquisition of mosaic *pbp2x, pbp2b, pbp1a*, and *murM* alleles, that their order of uptake influenced the accessible resistance trajectory, and that the final MIC varied with the recipient genome (Gibson et al., 2022). Similarly, interspecies recombinant strains could acquire β-lactam resistance and tolerance while retaining virulence, whereas resistance generated through *de novo* PBP mutation imposed greater *in vivo* fitness costs (Nishimoto et al., 2022). Thus, a PBP profile represents the product of a selected evolutionary route rather than a simple additive collection of resistance substitutions.

Although PBP variation forms the dominant predictive layer, the genetic architecture is not exclusively PBP based. The peptidoglycan-branching enzyme MurM can be required for clinically relevant amoxicillin resistance in specific allele combinations (Gibson et al., 2022), and genome-wide reconstruction experiments have identified additional cell-wall-associated determinants capable of increasing cefotaxime resistance, with many effects appearing lineage dependent (Fani et al., 2013). Epistasis and compensatory evolution also shape the expressed phenotype: the fitness and cell-division defects introduced by resistant *pbp2b* alleles can be offset by subsequent alterations in *pbp2x* and *pbp1a* (Albarracín Orio et al., 2011). More recently, loss-of-function variation in the cyclic-di-AMP phosphodiesterase gene *pde1* was shown to confer low-level penicillin resistance independently of classical PBP modification and potentially provide an evolutionary gateway toward higher resistance (Kobras et al., 2023). Collectively, these findings explain why PBP profiles are powerful but imperfect MIC predictors: they capture the principal target-based resistance architecture, while recipient background, non-PBP loci, epistasis, and compensatory changes account for part of the residual phenotypic variation.

## 3 From PBP profiles to genome-predicted MICs

### 3.1 PBP subtype- and profile-based prediction

The first practical framework for genome-based β-lactam susceptibility prediction converts the transpeptidase-domain sequences of PBP1a, PBP2b, and PBP2x into discrete sequence identifiers and then combines them into an ordered PBP profile. Terminology varies across studies: the foundational CDC-associated studies referred to the three-PBP combination as a “PBP type, “ whereas this review uses “PBP subtype” for an individual PBP sequence and “PBP profile” for their combination. In its most transparent form, profile-based prediction is a reference-table approach: an isolate with a previously characterized profile is assigned the modal MIC observed among isolates carrying that profile. Using 2, 528 invasive pneumococcal isolates, Li et al. identified 307 PBP profiles and showed that the combined PBP sequences explained 94–99% of MIC variation across penicillin, amoxicillin, meropenem, cefotaxime, ceftriaxone, and cefuroxime. For characterized profiles, modal-MIC predictions achieved >98% essential agreement, defined as agreement within one doubling dilution, and >94% categorical agreement with broth microdilution MICs (Li et al., 2016). The approach is readily interpretable and drug specific, but its confidence is inherently tied to the number and diversity of phenotyped isolates supporting each profile. This limitation is illustrated by a simple BLAST-based implementation: predictions were highly concordant for recognized pneumococcal profiles, but agreement declined when the nearest recognized profile was used for novel combinations (Eriksen et al., 2023).

### 3.2 Statistical and machine-learning prediction

Sequence-based statistical models extend prediction beyond exact profile matching by learning the contributions of individual amino-acid positions or resistance determinants. In the original PBP-typing framework, random forest and elastic-net models were evaluated alongside the modal-MIC lookup method; unlike exact matching, these models could generate estimates for previously uncharacterized profiles (Li et al., 2016). Subsequent validation using 4, 309 isolates from two datasets showed that random forest predictions for newly encountered profiles achieved >97% essential agreement and >93% categorical agreement for all six β-lactams, outperforming the modal-MIC approach for these profiles (Li et al., 2017). Alternative models have pursued different prediction targets. Zhang et al. used supervised learning based on fragments of *pbp2x* and *pbp2b* to predict amoxicillin and cefuroxime resistance phenotypes, demonstrating that reduced sequence inputs can support drug-specific categorical classification (Zhang et al., 2020). By contrast, Demczuk et al. constructed multivariable linear regression equations from molecular resistance determinants to predict numerical MICs. When applied to independent Canadian and US validation datasets, predictions within one doubling dilution reached 97.4% for penicillin and 98.2% for ceftriaxone (Demczuk et al., 2021). These approaches are therefore not interchangeable: profile lookup, regression, and classification models may output a modal MIC, a continuous or discretized MIC estimate, or a susceptibility category. Performance should consequently be evaluated against the intended output rather than summarized by a single accuracy measure.

### 3.3 Contemporary tools and scalable pipelines

The operational value of these models depends on their integration into reproducible workflows that accept routine WGS data and return standardized susceptibility outputs. An early CDC pipeline applied WGS-based resistance prediction to 2, 316 invasive isolates and showed low categorical discrepancy rates across multiple antimicrobials. For β-lactams, fourfold or greater differences between predicted and broth-microdilution MICs occurred in only 0.2–1.0% of isolates, and phenotypic retesting resolved a proportion of the initial discrepancies (Metcalf et al., 2016). More recently, Pathogenwatch and AREScloud were directly compared with conventional AST for 538 pneumococcal isolates. Both tools generated β-lactam MIC predictions and achieved >94% categorical agreement, with no or only one major or very major error for the evaluated β-lactams (Sanchez et al., 2024). The GPS Pipeline further embeds the CDC PBP AMR Predictor within a portable, containerized workflow that simultaneously reports serotype, lineage, and antimicrobial susceptibility. It predicts MICs for six β-lactams and converts them into categories according to the 2014 CLSI guideline. Among 1, 273 genomes with corresponding broth-dilution MICs for all six β-lactams, mean categorical concordance was 94.36%, although concordance for penicillin was lower at 88.22% (Hung et al., 2025).

Recent target-level and in silico studies provide complementary evidence that such outputs should remain drug-specific. Live-cell profiling of 16 β-lactams in *S. pneumoniae* D39 showed distinct PBP selectivity, with most compounds co-selective for PBP2x and PBP3, six selective for PBP3, and one co-selective for PBP1a and PBP3 (Sharan and Carlson, 2022). Structural and molecular-dynamics analyses of β-lactam-derived probes bound to pneumococcal PBP1b further demonstrated probe-specific binding modes (Flanders et al., 2022). Recent PBP2x-focused virtual screening studies using molecular docking and molecular dynamics also support the structural relevance of active-site or mutant-context interactions, while remaining exploratory and requiring phenotypic validation (Aribisala et al., 2024; Panickar et al., 2025). Collectively, these developments mark a transition from proof-of-concept genotype–phenotype association to scalable genomic susceptibility prediction, but they also indicate that predicted MICs should not be generalized as a universal β-lactam susceptibility category.

Representative approaches and tools for genome-based prediction of pneumococcal β-lactam susceptibility are summarized in **Table 1**.

**Table 1:** Representative approaches for genome-based prediction of β-lactam susceptibility in *Streptococcus pneumoniae*.

Study or tool	Genomic input	Prediction approach	Primary output	Validation scope	Reported performance	Main strength	Main limitation
Li et al., 2016 (Li et al., 2016)	PBP1a, PBP2b, and PBP2x transpeptidase-domain sequences	Exact PBP-profile lookup; random forest; elastic-net modeling	Drug-specific modal or predicted MICs for six β-lactams	2, 528 invasive pneumococcal isolates; 307 PBP profiles	>98% essential agreement and >94% categorical agreement for characterized profiles	Established the foundational PBP-profile prediction framework	Exact lookup depends on previously characterized profiles and phenotype-linked reference data
Li et al., 2017 (Li et al., 2017)	Newly encountered PBP1a/PBP2b/PBP2x profiles	Random forest prediction compared with modal-MIC assignment	Predicted MICs for six β-lactams	4, 309 isolates from two datasets; focused on previously uncharacterized profiles	>97% essential agreement and >93% categorical agreement for all six β-lactams	Demonstrated prediction beyond exact profile matching	Novel substitutions and rare combinations outside the training space remain uncertain
Metcalf et al., 2016 (Metcalf et al., 2016)	Whole-genome sequences and known resistance determinants	Integrated CDC WGS-based resistance prediction workflow	Predicted MICs and susceptibility categories	2, 316 invasive pneumococcal isolates collected in the United States	Fourfold-or-greater β-lactam MIC discrepancies occurred in only 0.2–1.0% of isolates	Demonstrated feasibility for large-scale public-health surveillance	Validation population was dominated by a single national surveillance system
Zhang et al., 2020 (Zhang et al., 2020)	Selected fragments of *pbp2x* and *pbp2b*	Supervised machine-learning classification	Drug-specific resistance phenotype	Amoxicillin- and cefuroxime-focused classification datasets	Showed that reduced PBP sequence inputs can support drug-specific categorical prediction	Computationally efficient and focused on parsimonious sequence features	Limited drug scope and categorical output; does not directly provide full MIC distributions
Demczuk et al., 2021 (Demczuk et al., 2021)	Molecular antimicrobial-resistance determinants	Multivariable linear-regression equations	Numerical MIC predictions	Independent Canadian and US validation datasets	Predictions within one doubling dilution reached 97.4% for penicillin and 98.2% for ceftriaxone	Provides explicit numerical MIC estimates and interpretable regression equations	Performance depends on the completeness and stability of included resistance determinants
Eriksen et al., 2023 (Eriksen et al., 2023)	PBP-profile sequences from *S. pneumoniae* and other mitis-group streptococci	BLAST-based exact or nearest-profile matching	Predicted MICs and susceptibility categories	Recognized and novel pneumococcal profiles, plus non-pneumococcal mitis-group isolates	High concordance for recognized pneumococcal profiles; accuracy declined for novel combinations and other species	Transparent and readily implementable reference-matching approach	Nearest-profile assignment may overstate confidence for novel, recombinant, or non-pneumococcal sequences
Pathogenwatch and AREScloud (Sanchez et al., 2024) (Sanchez et al., 2024)	Routine whole-genome sequencing data	Integrated genomic susceptibility-prediction tools	β-lactam MIC predictions and susceptibility categories	538 pneumococcal isolates directly compared with conventional AST	>94% categorical agreement; no or only one major or very major error for evaluated β-lactams	Direct contemporary comparison with routine phenotypic AST	Outputs depend on tool-specific databases, models, and breakpoint implementations
GPS Pipeline (Hung et al., 2025) (Hung et al., 2025)	Whole-genome sequencing data	Portable, containerized pipeline incorporating the CDC PBP AMR Predictor	MICs and categories for six β-lactams, together with serotype and lineage	1, 273 genomes with broth-dilution MICs for all six β-lactams	Mean categorical concordance 94.36%; penicillin concordance 88.22%	Scalable, reproducible integration of susceptibility prediction with genomic surveillance	Uses fixed model and breakpoint versions; performance varies by antimicrobial agent

AST, antimicrobial susceptibility testing; MIC, minimum inhibitory concentration; PBP, penicillin-binding protein; WGS, whole-genome sequencing.

## 4 Why PBP-based predictions do not always generalize

### 4.1 Novel and sparsely represented PBP profiles

Profile-based MIC assignment is fundamentally an empirical association rather than a mechanistic guarantee. Its most reliable application is therefore an exact PBP-profile match supported by multiple isolates with concordant reference MICs. When an exact match is unavailable, nearest-profile approaches assume that sequence similarity corresponds to functional equivalence, whereas statistical models extrapolate from substitutions and combinations represented in their training data. In a validation dataset, random forest models predicted MICs more accurately than modal-MIC assignment for 148 isolates carrying 109 previously uncharacterized PBP profiles; nevertheless, substitutions absent from the training data remained a recognized source of uncertainty (Li et al., 2017). Experimental reconstruction of amoxicillin resistance further supports this concern: clinically relevant resistance required ordered acquisition of mosaic *pbp2x, pbp2b, pbp1a*, and *murM* alleles, and the resulting MIC depended on both the order of acquisition and the recipient genome (Gibson et al., 2022). Continued pneumococcal evolution ensures that such profiles are not exceptional. A Japanese study identified 21 novel PBP profiles among 71 nonencapsulated pneumococci, while an emerging amoxicillin- and meropenem-resistant 23A-ST166 lineage in Taiwan predominantly carried the novel profile 15:11:299 (Kawaguchiya et al., 2022; Chen et al., 2023). Consequently, prediction systems should distinguish exact profile matches from model-based extrapolations and profiles lacking an adequately represented analogue, rather than presenting all predicted MICs with equivalent confidence.

### 4.2 Interspecies recombination and the mitis-group gene pool

The sequence space relevant to pneumococcal β-lactam susceptibility extends beyond the pneumococcal population itself. Closely related mitis-group streptococci share homologous cell-wall synthesis genes with *S. pneumoniae* and can act as donors of divergent *pbp* fragments. Comparative genomic analyses identified *Streptococcus mitis* as a frequent donor of PBP-associated sequences and showed that pneumococci acquiring such fragments tended to exhibit higher penicillin MICs; the probability of receiving fragments also differed among pneumococcal serotypes (Kalizang'oma et al., 2021). Interspecies homologous recombination has similarly contributed to the independent emergence and geographical diversification of multidrug-resistant pneumococcal lineages (D'Aeth et al., 2021). Importantly, the biological relevance of recombinant resistance is supported by *in vivo* evidence: serially transformed recombinant pneumococci acquired penicillin resistance and tolerance while retaining virulence in mice, whereas resistance generated through *de novo* PBP mutation imposed greater fitness and virulence costs (Nishimoto et al., 2022). The consequences for prediction are illustrated by applying a pneumococcal PBP-profile database to other mitis-group streptococci: none of the non-pneumococcal isolates carried a recognized profile, and categorical agreement varied substantially by species and drug (Eriksen et al., 2023). Although clinical prediction is ordinarily performed after species identification, imported recombinant segments can still introduce sequence configurations outside the reference database. Prediction workflows therefore need to detect highly divergent or recombinant alleles and avoid overconfident assignment to the nearest known pneumococcal profile.

### 4.3 Lineage and population dependence

PBP profiles are embedded within a strongly structured pneumococcal population. Their distribution is associated with clonal lineage, serotype, geography, antimicrobial exposure, and vaccine-driven population change. Models developed and internally validated using datasets dominated by the same successful resistant clones may consequently appear more transferable than they are. In an international analysis of 20, 027 genomes, 621 Global Pneumococcal Sequence Clusters were defined, and the country of isolation significantly predicted the antibiogram within 28 of the 35 dominant clusters, demonstrating substantial geographical structure even within widely distributed lineages (Gladstone et al., 2019). More recent global resources, including the Pneumococcal Genome Library containing 30, 976 genomes from approximately 80 countries, substantially broaden the representation of pneumococcal genomic diversity (Jansen van Rensburg et al., 2024). Contemporary pipelines also increasingly integrate serotype, lineage, and susceptibility prediction within the same workflow, providing an opportunity to evaluate PBP-based predictions in their population context rather than as isolated sequence profiles (Hung et al., 2025).

This dependence should be addressed by structured validation and confidence stratification rather than by assuming a single globally transferable performance estimate. External validation should be stratified by serotype, ST, GPSC, geographic region, collection period, profile frequency, and profile novelty. Where possible, prediction performance should also be evaluated under leave-one-region, leave-one-lineage, or temporally separated validation designs to test whether accuracy is preserved outside the dominant training populations. Recent clinical epidemiological data further illustrate why such stratification is relevant: amoxicillin non-susceptibility in invasive pneumococcal pneumonia can be concentrated within specific lineage-serotype backgrounds, such as GPSC6 expressing serotypes 9V, 14, or 11A (Càmara et al., 2025). Therefore, locally dominant lineages, emerging serotype-switched clones, and underrepresented PBP profiles should be flagged for cautious interpretation and phenotypic confirmation until sufficient standardized MIC data have accumulated in the relevant population.

### 4.4 Phenotypic reference uncertainty

Genomic prediction models learn and are evaluated against phenotypic MICs; therefore, their apparent accuracy is partly constrained by the quality and consistency of the phenotypic reference. Pneumococcal β-lactam MIC results may differ among testing platforms, particularly near interpretive breakpoints. In a comparison of four commercial methods with broth microdilution, essential agreement was at least 90% for most method–drug combinations, but ceftriaxone categorical agreement was only 68–88% for several methods, with very major error rates of 6–12% when meningitis breakpoints were applied (Charles et al., 2015). A recent direct comparison of Pathogenwatch and AREScloud with conventional AST nevertheless demonstrated greater than 94% categorical agreement for β-lactams and very few major or very major errors under defined validation conditions (Sanchez et al., 2024). Discordant genotype–phenotype results should therefore prompt review of sequence quality, profile assignment, testing methodology, and, where necessary, repeat reference MIC testing rather than being attributed automatically to model failure.

## 5 From predicted MICs to potential clinical interpretation

The preceding sections show that PBP profiles capture the principal genetic architecture of pneumococcal β-lactam susceptibility and can support accurate MIC prediction at population scale. However, prediction of a technically credible MIC is not the endpoint of clinical antimicrobial susceptibility testing. Potential clinical interpretation begins only when that predicted MIC is interpreted using an appropriate breakpoint and within a defined therapeutic context.

### 5.1 A predicted MIC is not a context-independent susceptibility category

Clinical breakpoints are decision thresholds derived by integrating MIC distributions, resistance mechanisms, pharmacokinetic and pharmacodynamic exposures, dosing regimens, and available clinical outcome data; they do not represent intrinsic properties encoded solely by the bacterial genome (Turnidge and Paterson, 2007; Mouton et al., 2012). Pneumococcal penicillin susceptibility provides a particularly clear example. The same penicillin MIC may receive different categorical interpretations depending on whether treatment is intended for meningitis, a non-meningeal infection treated with parenteral penicillin, or an infection treated orally. The revised CLSI penicillin breakpoints were explicitly developed to distinguish these therapeutic settings, and epidemiological studies have shown that applying different breakpoint schemes can markedly alter reported susceptibility and resistance rates without any change in the underlying MIC distribution (Weinstein et al., 2009; Imöhl et al., 2014; Alford et al., 2023).

Interpretation must also remain drug specific. Although related β-lactams may share major PBP determinants, a predicted MIC or susceptibility category for one agent should not automatically be extrapolated to another. A recent reevaluation showed that the oral penicillin susceptible breakpoint predicted cefpodoxime and ceftriaxone susceptibility, whereas cefpodoxime susceptibility could not be inferred reliably among isolates nonsusceptible by the oral penicillin breakpoint, with categorical agreement falling to 78.4% (Mendes et al., 2025). Parenteral penicillin breakpoints also performed poorly for cefpodoxime prediction, with a categorical agreement of 76.8%, although they retained value as a surrogate marker for ceftriaxone susceptibility (Mendes et al., 2025). Earlier analysis of 249 pneumococcal isolates similarly showed that, among 240 isolates categorized as cefotaxime susceptible using CLSI non-meningitis breakpoints, 23% would have been misrepresented as cefdinir susceptible; by contrast, amoxicillin and penicillin categories were discordant in only one isolate (Murphy et al., 2021). Accordingly, a PBP-based prediction should be treated as an estimate for a specified β-lactam rather than as a universal label of “β-lactam resistance” or “penicillin resistance”.

Clinical outcome data further support this context-dependent approach. In a recent observational study of 1, 663 episodes of invasive pneumococcal pneumonia, amoxicillin non-susceptibility was concentrated in a single lineage, GPSC6 expressing serotypes 9V, 14, and 11A, which accounted for 56.5% of non-susceptible strains (Càmara et al., 2025). Amoxicillin therapy was associated with higher 30-day mortality than third-generation cephalosporin therapy, particularly in episodes caused by isolates with amoxicillin MIC >2 mg/L; however, the association also varied according to host prognosis (Càmara et al., 2025). These findings indicate that PBP-derived MIC prediction may contribute to potential clinical interpretation, but only when interpreted together with the β-lactam agent, infection site, route or exposure context, breakpoint standard, local lineage structure, and patient-level clinical context.

### 5.2 A three-layer framework for genomic susceptibility reporting

To preserve the distinction between genomic evidence, predicted phenotype, and therapeutic interpretation, we propose a three-layer reporting framework. The first layer should describe the genomic finding itself, including the assigned PBP1a, PBP2b, and PBP2x subtypes, their combined PBP profile, and whether the result represents an exact match to a phenotypically characterized profile, a model-based extrapolation, or an insufficiently represented sequence combination. Relevant sequence-quality limitations and evidence of highly divergent or recombinant alleles should also be disclosed.

The second layer should report the predicted phenotype. This may consist of a predicted MIC, an MIC interval, or a categorical prediction, but should always identify the antimicrobial agent, prediction model, reference database and version, and the confidence or evidential support underlying the estimate. Proximity to a clinical breakpoint is particularly important because an error of one doubling dilution may have little practical consequence when the MIC lies far from a breakpoint but may reverse the susceptibility category when the estimate lies close to it. Contemporary tools such as Pathogenwatch, AREScloud, and the GPS Pipeline demonstrate that standardized genomic susceptibility outputs can be generated at scale; nevertheless, their reported categories remain dependent on the incorporated model, database, and breakpoint criteria (Sanchez et al., 2024; Hung et al., 2025).

**Figure 1:**
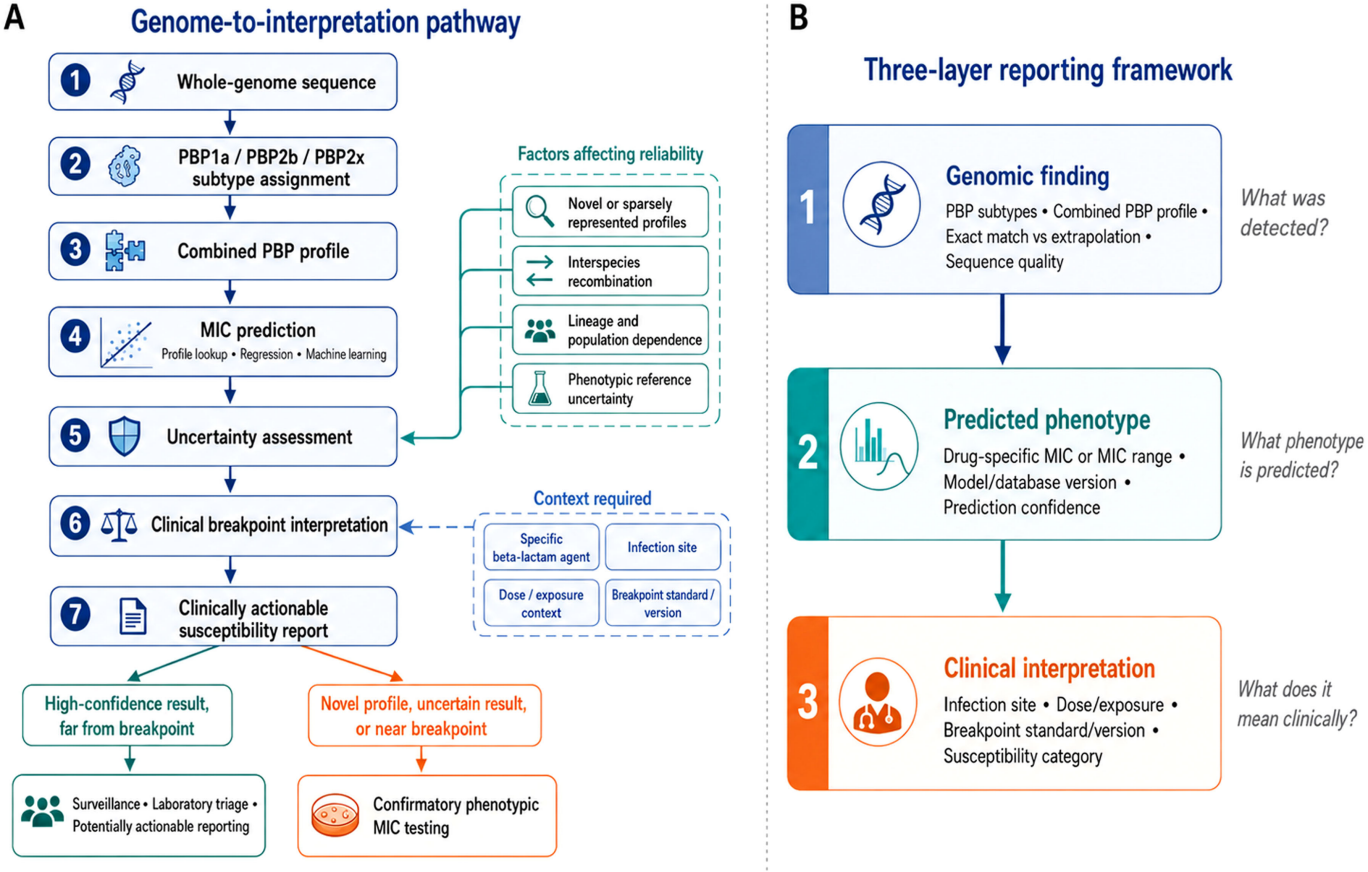
From pneumococcal PBP profiles to potential clinical interpretation of β-lactam susceptibility. **(A)** Genome-to-interpretation pathway showing PBP subtype assignment, combined PBP profiling, MIC prediction, uncertainty assessment, breakpoint interpretation, and indications for phenotypic confirmation. **(B)** Three-layer reporting framework separating genomic findings, predicted phenotypes, and clinical interpretation. MIC, minimum inhibitory concentration; PBP, penicillin-binding protein.

The third layer is the potential clinical interpretation. A categorical result should be generated only after specifying the antimicrobial agent, infection site, relevant route or level of antimicrobial exposure, interpretive standard, and breakpoint version. Differences among clinical settings should therefore be managed through context-stratified interpretation rather than by applying a single universal susceptibility category. For population surveillance, applying a fixed and explicitly documented interpretive framework may be desirable to preserve comparability over time. For patient-level reporting, however, the interpretation must reflect the current locally adopted standard and the clinical context of the infection. A stand-alone statement such as “penicillin resistant, “ without specifying the applicable breakpoint context, therefore conveys incomplete and potentially misleading information. The proposed pathway from PBP profiling to potential clinical interpretation of β-lactam susceptibility is summarized in **Figure 1**.

### 5.3 When phenotypic confirmation remains necessary

Phenotypic MIC testing should remain the confirmatory approach when a genomic result falls outside the validated scope of the prediction system or when the consequences of misclassification are substantial. Confirmation should be prioritized for isolates carrying novel or sparsely represented PBP profiles, sequences of uncertain quality, highly divergent or recombinant alleles, and predicted MICs within one doubling dilution of a relevant breakpoint. It is also particularly important for meningitis and other high-consequence infections, for β-lactams or dosing contexts not independently validated by the model, and whenever genomic and available phenotypic findings are discordant.

Conversely, an exact and well-supported PBP-profile match predicting an MIC far from the relevant breakpoint may provide robust evidence for surveillance, isolate prioritization, and laboratory triage. However, patient-level reporting still requires external validation in the intended population and predefined acceptable rates of categorical, major, and very major errors. A recent comparison with conventional AST confirms high overall categorical agreement, while showing that performance must be assessed separately for each drug, population, testing method, and breakpoint framework (Sanchez et al., 2024). Genome-based susceptibility interpretation is therefore an additional layer integrating genomic evidence, prediction uncertainty, phenotypic validation, and treatment-specific breakpoints.

## 6 Discussion: a pathway toward potential clinical interpretation

Genome-based prediction of pneumococcal β-lactam susceptibility has progressed from proof-of-concept PBP–MIC associations to scalable pipelines capable of generating standardized susceptibility predictions. Recent evaluations demonstrate high overall agreement with conventional antimicrobial susceptibility testing. However, aggregate accuracy alone is insufficient for patient-level interpretation, because clinical reliability also depends on population representativeness, reference MIC quality, prediction uncertainty, and the interpretive context applied to each result (Sanchez et al., 2024; Hung et al., 2025).

Several developments are required before genome-predicted MICs can support potential clinical interpretation in patient-level contexts. First, PBP–MIC reference resources should be globally representative, continuously updated, strictly curated, and version controlled. Each profile should be linked to standardized phenotypic MICs, testing methods, isolate metadata, and the number of independently characterized isolates supporting the association. Second, prediction systems require external validation across geographical regions, pneumococcal lineages, collection periods, and clinical settings. Performance should be stratified by antimicrobial agent, profile novelty, MIC proximity to relevant breakpoints, and intended use, rather than summarized solely by overall categorical agreement. Third, reporting systems should communicate uncertainty explicitly and define prospective criteria that trigger confirmatory phenotypic testing. International recommendations for WGS-based susceptibility prediction have similarly emphasized quality-control metrics, curated databases, reference-method phenotypes, and validation within the intended application (Ellington et al., 2016; Samuelsen et al., 2026).

Implementation should distinguish population surveillance from patient-level decision-making. Genomic prediction is already well positioned to strengthen surveillance, characterize emerging resistant lineages, and prioritize isolates for phenotypic investigation. Direct clinical reporting additionally requires integration with current local breakpoints, laboratory quality-management systems, acceptable turnaround times, and prospective evidence of clinical utility. Effective translation will therefore depend on sustained linkage among genomic surveillance, reference laboratories, clinical microbiology services, and phenotype-linked databases (Baker et al., 2023; Sherry et al., 2025). PBP-based prediction is sufficiently mature to support increasingly informative surveillance and may inform potential clinical interpretation, but patient-level use requires an uncertainty-aware interpretive layer connecting genomic evidence to validated phenotypes and treatment-specific breakpoints.
